# Gay men’s stress response to a general and a specific social stressor

**DOI:** 10.1007/s00702-021-02380-6

**Published:** 2021-07-27

**Authors:** Frank A. Sattler, Urs M. Nater, Ricarda Mewes

**Affiliations:** 1 Department of Psychology, Clinic for Orthopedic Rehabilitation, Klinik am Homberg, Hans-Georg-Weg 2, 34537 Bad Wildungen, Germany; 2 Department of Clinical and Health Psychology, Faculty of Psychology, University of Vienna, Liebiggasse 5, 1010 Wien, Austria; 3 Research Platform ‘The Stress of Life (SOLE)’, University of Vienna, Liebiggasse 5, 1010 Wien, Austria; 4 Outpatient Unit for Research, Teaching and Practice, Faculty of Psychology, University of Vienna, Renngasse 6-8, 1010 Wien, Austria

**Keywords:** Gay men, Salivary cortisol, Salivary testosterone, Perceived stress, Gay-specific stress test, Experimental study

## Abstract

Gay men show altered psychobiological stress responses and exhibit a higher prevalence of mental disorders than their heterosexual counterparts. Both of these findings are likely due to gay-specific discrimination. Since it has not yet been determined whether gay-specific stress is more noxious than general stress, we tested whether gay men react more strongly to gay-specific socially stressful stimuli than to general socially stressful stimuli. *N* = 33 self-identified gay men (mean = 26.12 years of age, SD = 5.89), 63.6% of whom were in a relationship with a man, participated in an experimental within-group study, in which they were exposed to the Trier Social Stress Test (TSST) as well as a gay-specific TSST in a randomized order. Salivary cortisol and testosterone were assessed at five time points during the laboratory tests and perceived stress was assessed at four time points. According to psychobiological and perceived stress indices, the participants reacted similarly to a gay-specific and general social stressor. There were no significant differences in the outcomes, either when looking at pre–post-test differences or when comparing the overall stress responses. Given that the response to a gay-specific social stressor was equally pronounced as the one to a general social stressor, programs aiming to decrease minority stress but overlooking general stress are likely to yield only partial improvements in gay men’s mental health. Instead, we suggest helping gay men cope with both forms of stress through building social support, assertiveness, and mindfulness skills, as well as decreasing emotional dysregulation.

## Introduction

Gay men constitute a risk group for physical (Branström et al. 2016) as well as mental disorders (Plöderl and Tremblay 2015; Ross et al. 2018; Semlyen et al. 2016) and show a higher mortality rate compared to heterosexual men (Cochran et al. 2016).

Meyer (2003) suggested that gay men experience gay-specific stressors, also called minority stressors, which explain mental and physical health differences compared to heterosexual men. Minority stressors include gay-related structural stigma (Hatzenbuehler et al. 2020), gay-related victimization (Pachankis 2015), expectations of gay-related rejection (Pachankis 2015), and internalized homonegativity (Berg et al. 2016), which refers to the tendency of some gay men to devalue themselves because of their sexual orientation. In addition, studies have found that gay men and other sexual minorities are more likely than heterosexual individuals to experience early-life adversity (McLaughlin et al. 2012) and (general) stressful life events, such as debt or termination of personal relationships (Przedworski et al. 2015).

According to Cohen et al. (2016), the stress process starts with a stressor that may be appraised as either stressful or not. Perceived stress then sets off a cascade of negative emotions, poor health decisions and behaviors, and an activation of the two major stress-responsive systems, the sympathoadrenal-medullary (SAM) system as well as the hypothalamic–pituitary–adrenal (HPA) axis (Cohen et al. 2016). In the case of acute stressors, the body responds with short-term adaptations, including adjustment of blood pressure, cortisol, and testosterone secretion (McEwen 2004). However, if the stressor is chronic or prolonged, the bodily stress responses are inefficiently turned on and off repeatedly, leading to wear and tear of the body; a harmful process called allostatic load (Coffman 2020; McEwen and Seeman 1999). This is associated with disease-related physiological changes (e.g., immunological and cardiovascular ones) and an increased risk of disease onset or progression (Cohen et al. 2016). In the current study, we combine the psychobiological and psychosocial perspectives on stress, taking into account both physiological as well as perceived stress outcomes.

In an experimental study, lesbians, gay men, and bisexual (LGB) individuals were exposed to an LGB-related version of the Trier Social Stress Test (TSST) (Kirschbaum et al. 1993), which included talking about an event in which they felt rejected based on their sexual orientation in front of two interviewers (Hatzenbuehler and McLaughlin 2014). The researchers found that those individuals who had spent their adolescence in US states with high LGB-directed structural stigma showed blunted stress responses compared to those who had lived in states with low structural stigma (Hatzenbuehler and McLaughlin 2014). Another laboratory study using the original version of the TSST found that gay and bisexual men showed a lower overall salivary cortisol concentration from baseline to post-TSST than did heterosexual men (Juster et al. 2015). However, studies looking at diurnal cortisol patterns found that LGB and heterosexual individuals did not differ in diurnal cortisol secretion (Austin et al. 2016; Juster et al. 2013). The overall findings thus indicate that gay men have normal diurnal cortisol patterns, but when exposed to an acute stressor, they exhibit hypocortisolism, a factor associated with allostatic load (Fries et al. 2005).

A further psychobiological variable that changes in response to stress is salivary testosterone: while testosterone is decreased by chronic stress, short-term testosterone increases can be expected when men are exposed to an acute stressor (Afrisham et al. 2016). A study targeting HIV-seropositive gay and bisexual men, who due to HIV tended to have diminished levels of testosterone, found that a cognitive-behavioral stress management intervention led not only to reduced distress but also to an increase in free testosterone in the blood, which was in turn related to a decrease in anxiety and depressive symptoms (Cruess et al. 2000). However, we are unaware of any studies that have tested for testosterone changes in response to an acute stressor in gay men.

The objective of our study was to compare how gay men respond to general and gay-specific stressors and whether these stress responses differ. It is important to gain insight into this question, as it could improve prevention and intervention strategies for gay men. For instance, while clinical interventions have tackled minority stress rather specifically (Pachankis 2015; Proujansky and Pachankis 2017) or have applied a somewhat broader scope of decreasing stress in general (Pepping et al. 2017), it remains unclear whether general or gay-related stressors are more relevant to gay men’s health. We expect that:

(1) Compared to their response to an (acute) general stressor, gay men will show a blunted direct response to an (acute) gay-related stressor with respect to salivary cortisol, salivary testosterone, and perceived stress; (2) gay men will show lower overall levels of salivary cortisol, salivary testosterone, and perceived stress throughout the whole session when exposed to an (acute) gay-related stressor as compared to an (acute) general stressor.

## Methods

### Participants

The data for the present study were collected as part of the project “Psychobiological Consequences of Discrimination for Gay Men” at the University of Marburg, Germany. The project was approved by the Ethics Committee of the Department of Psychology, University of Marburg (reference number 2016-30k). Participants were recruited via snowball sampling, mailing lists for university students and employees, and flyers in the university’s cafeterias. The subjects received 32 Euros for participating in the full study or—in the case of dropping out—8 Euros for every hour attended.

The study’s inclusion criteria were: (1) identifying as a gay man, (2) age 18 years or older, and (3) having at least one experience of gay-related discrimination. The third inclusion criterion was necessary since the participants were required to talk about gay-related discrimination during one of the stress tests. To screen for gay-related discrimination, we asked all participants “Have you ever experienced discrimination based on your sexual orientation?”.

A total of 36 self-identified gay men participated in the study between December, 2016 and January, 2018. Three participants dropped out after the first laboratory session and were therefore excluded from the analysis. The final sample consisted of *N* = 33 gay men, with a mean age of 26.12 years (SD = 5.89, range = 18 to 47 years). Most participants reported being in a relationship with a man (63.6%, *n* = 21). We did not ask for more specification (e.g., cohabitation status) or other types of relationships (e.g., relationship with a woman). The majority of the sample (84.8%, *n* = 28) reported being attracted only to men, with the remainder (15.2%, *n* = 5) being attracted mostly to men. As it has been noted earlier, it is not unusual that “as a result of social and cultural influences, sexual attractions and sexual orientation identity do not always correspond” (Sell 2007). Because of this and since our first inclusion criterion was based on self-identifying as a gay man only, the *n* = 5 participants who reported being attracted mostly to men were kept in the dataset for analysis. The participants' median highest educational attainment was a higher-track school-leaving qualification (60.6%, *n* = 20) and the median monthly salary level lay between 500 and 1000 Euros (36.4%, *n* = 12). 84.6% had experienced at least one form of gay-related discrimination in the last year.

### Measures

Saliva samples were collected via the passive drool method using SaliCaps (IBL International, a Tecan Group company, Hamburg, Germany). In brief, participants were asked to collect saliva in their mouth for 2 min and then transfer the cumulated saliva into a tube via a straw. Samples were stored at − 20 °C until analysis. Salivary cortisol and testosterone concentrations were determined using commercial enzyme-linked immunosorbent assays (ELISA; IBL International, a Tecan Group company, Hamburg, Germany), measured by a spectrometer (Spectrostar nano, BMG Labtech, Ortenberg, Germany).

Since it has been found earlier that a single-item stress measure has satisfactory content, criterion, and construct validity (Elo et al. 2003), we assessed momentary perceived stress with a one-item visual analogue scale (VAS), which has been used previously (Fischer et al. 2016; Linnemann et al. 2015). Thereby, the participants responded to the item “I feel stressed” on a VAS ranging from 1 (completely disagree) to 100 (completely agree).

To test whether the stress induction was successful, we included a manipulation check (MC), by asking “Do you feel this social situation was stressful?” on a VAS from 1 (completely disagree) to 100 (completely agree). To differentiate this VAS from the one used to measure momentary perceived stress, we call it VAS_MC_.

### Design

The current study employs a highly controlled experimental and laboratory design in which participants are tested in a general and gay-specific stress test, in a randomized order, with a 7-day lag between the two tests.

The TSST (Kirschbaum et al. 1993) was used to induce general social stress. A video camera and a voice recorder were installed in the room where the TSST was applied. The participants were told that the test consisted of a mock job selection interview for a job of their choice. They were given 10 min to prepare for a 5-min free speech in front of a committee consisting of a male and a female member (public-speaking task). After talking for 5 min, they were asked to complete a 5-min mental arithmetic task involving a continuous backwards subtraction from a 3-digit number (cognitive task). Previous researchers found that the TSST leads to an increase in several stress markers, among them salivary cortisol (Kirschbaum et al. 1993), and that public-speaking/cognitive tasks elicit the largest effect size in terms of stress induction among all commonly used stress tests (Dickerson and Kemeny 2004).

To induce gay-related social stress, we used a version of the TSST by Hatzenbuehler and McLaughlin (2014), which induces LGB-related stress by asking the participants to talk freely for 5 min about an experience in which they felt rejected because of their sexual orientation. Following this, participants completed the serial subtraction task described above. Since we only included gay men as participants, we termed this stress test Gay-Specific TSST (GS-TSST).

### Procedure

After completing an online screening questionnaire which asked for inclusion criteria and a preliminary online informed consent, participants were invited to two 2-h laboratory sessions with 7 days in between, which were scheduled somewhere between 2 and 7 pm to control for diurnal fluctuations in salivary cortisol and testosterone (Dabbs 1990; Ghiciuc et al. 2011). At the beginning of the first laboratory session, the participants provided written informed consent, in accordance with the Declaration of Helsinki.

At minutes 30 (t1) and 48 (t2), we collected saliva samples and the participants provided information about their perceived stress on the VAS (see Table 1 for an overview of the laboratory sessions). At minute 50, the participants were randomly exposed to either the TSST or the GS-TSST in a second room. When the test ended at minute 60, the participants were brought back to the original room, where the third assessment (t3), which included a salivary sample, the VAS, and the VAS_MC_, occurred. The final assessments took place at minutes 80 (t4), with a salivary sample, and 110 (t5), with both a salivary sample and VAS. To ensure that the stress induction would not lead to long-term negative effects (such as heightened negative emotions), we offered our participants a free consultation with a psychologist/psychotherapist after t5. Afterwards, they were dismissed. The second laboratory session was scheduled 7 days later at the same time of the day. This session followed the same procedure as the first one, with the only difference being that the participants received the social stress test to which they had not yet been exposed (TSST or GS-TSST). Note that none of the participants utilized the offer to meet with a psychologist/psychotherapist.

**Table 1:** Assessment procedure in laboratory sessions

	t1	t2	t3	t4	t5
Minute	30	48	60	80	110
Description	Baseline	Directly before social stress test	Directly after social stress test	20 min recovery	50 min recovery
Assessed variables	Saliva sample, VAS	Saliva sample, VAS	Saliva sample, VAS, VAS_MC_	Saliva sample	Saliva sample, VAS

VAS = visual analogue scale assessing perceived stress, VAS_MC_ = visual analogue scale for the manipulation check. The saliva samples were later assessed for cortisol and testosterone levels. We used the same assessment procedure in both laboratory sessions

### Data analysis

All analyses not otherwise specified were conducted in IBM Statistics SPSS 27. To test hypothesis 1, we computed a one-way repeated measures multivariate analysis of variance (MANOVA) using the TSST and GS-TSST as conditions and the following three outcome variables: t1 to t3 difference in salivary cortisol; t1 to t3 difference in salivary testosterone; and t1 to t3 difference in perceived stress. The t1 to t3 differences, which measure the stress response to the stress test, were calculated by subtracting the t1 score from the t3 score.

To test the related hypothesis 2, we computed a one-way repeated measures MANOVA with two conditions (TSST and GS-TSST) and the following three outcomes: area under the curve with respect to the ground (AUC_G_) for salivary cortisol; AUC_G_ for salivary testosterone; and AUC_G_ for perceived stress. AUC_G_ was computed using the formula by Pruessner et al. (2003). It included all five time points (t1, t2, t3, t4, and t5) in the case of salivary cortisol and salivary testosterone and all four time points in the case of perceived stress (t1, t2, t3, and t5; compare Table 1).

A significance level of *p* < 0.05 was used. Marginally significant findings at *p* < 0.10 are pointed out in the results section but omitted in the discussion section due to their low evidential value. Cohen’s *d* was computed for all comparisons. In addition, we computed post hoc power analyses for our main analyses with G*Power version 3.1.9.6.

## Results

### Descriptive results

**Fig. 1:**
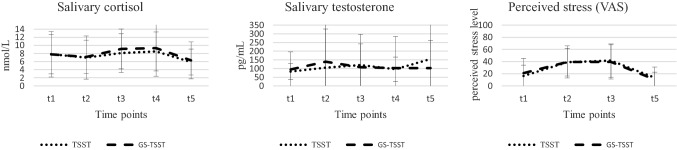
Means and standard deviations of outcomes at different time points. TSST = Trier Social Stress Test, GS-TSST = Gay-Specific TSST, VAS = visual analogue scale. Perceived stress was not assessed at t4

The means and standard deviations of our outcome variables are displayed in Table 2 and Fig. 1. Descriptively, the participants’ levels of salivary cortisol decreased from t1 to t2 in both conditions (TSST and GS-TSST), indicating an adaptation to the laboratory environment.

**Table 2:** Means and standard deviations of outcomes at t1 to t5

Time point	C		T		VAS	
	TSST	GS-TSST	TSST	GS-TSST	TSST	GS-TSST
t1	7.81 (4.84)	7.80 (5.60)	82.68 (46.11)	94.53 (101.43)	16.47 (17.67)	20.94 (24.23)
t2	7.00 (5.33)	7.15 (4.13)	105.52 (221.85)	139.29 (241.77)	39.00 (22.68)	39.47 (26.36)
t3	8.09 (4.77)	9.12 (4.91)	119.57 (177.23)	108.03 (133.53)	41.63 (27.35)	39.49 (28.18)
t4	8.50 (4.80)	9.32 (6.95)	95.56 (70.17)	102.76 (181.68)	–	–
t5	5.88 (3.20)	6.29 (4.55)	154.67^1^ (486.09)	102.33 (160.25)	13.54 (17.43)	10.49 (12.05)

C = salivary cortisol in nmol/L, T = salivary testosterone in pg/mL, VAS = visual analogue scale assessing perceived stress; numbers display means, numbers in parentheses display standard deviations. VAS was not assessed at t4. ^1^This high value is due to one outlier; if we delete it, the value is 70.47 (SD = 49.24). We decided to nevertheless include it since the main results would not change significantly after exclusion

At t3, the participants rated the TSST in the VAS_MC_ as stressful at 56.35 (SD = 28.17) and the GS-TSST at 55.09 (SD = 24.12). In addition, Table 2 shows that salivary cortisol and perceived stress increased from t2 to t3, providing further indication that the manipulation was successful. In the case of salivary testosterone, we found an increase in the TSST condition, but a drop in the GS-TSST condition from t2 to t3. Overall, these findings suggest that both the TSST and GS-TSST were successful in inducing stress.

We found that the participants had descriptively lower levels of salivary testosterone and perceived stress at t3 and t5 when exposed to the GS-TSST in comparison to the TSST. However, the salivary cortisol level was descriptively higher at t3, t4, and t5 in the GS-TSST than TSST condition, as was the salivary testosterone level at t4.

### Hypothesis 1: compared to their response to an (acute) general stressor, gay men will show a blunted direct response to an (acute) gay-related stressor with respect to salivary cortisol, salivary testosterone, and perceived stress

The one-way repeated measures MANOVA revealed a marginally significant effect for the overall comparison between the conditions TSST versus GS-TSST, *F*(3, 29) = 2.80, *p* < 0.10, *d* = 1.08. However, the univariate comparisons for salivary cortisol, *F*(1, 31) = 1.88, *p* > 0.05, *d* = 0.49, salivary testosterone, *F*(1, 31) = 0.83, *p* > 0.05, *d* = 0.33, and perceived stress, *F*(1, 31) = 2.86, *p* > 0.05, *d* = 0.61, between the two conditions were all non-significant. The post hoc power (1-*β*) for the comparison of salivary cortisol was 0.62, the one of salivary testosterone 0.71, and the one of perceived stress 0.85.

The findings do not support hypothesis 1, but indicate that gay men’s response in salivary cortisol, testosterone secretion, and perceived stress was similar when exposed to gay-related and general social stressors.

### Hypothesis 2: gay men will show lower overall levels of salivary cortisol, salivary testosterone, and perceived stress throughout the whole session when exposed to an (acute) gay-related stressor as compared to an (acute) general stressor

The AUC_G_ means and standard deviations of all measures in both conditions are shown in Table 3. The overall effect of the one-way repeated measures MANOVA was not significant, *F*(3, 23) = 0.78, *p* > 0.05, *d* = 0.64. In addition, the univariate tests comparing salivary cortisol, *F*(1, 25) = 1.20, *p* > 0.05, *d* = 0.44, salivary testosterone, *F*(1, 25) = 0.13, *p* > 0.05, *d* = 0.14, and perceived stress, *F*(1, 25) = 0.30, *p* > 0.05, *d* = 0.22, between the conditions were non-significant. This indicates that the overall psychobiological and perceived stress levels did not differ between the two experimental conditions (TSST and GS-TSST). In the post hoc power analyses, the comparison of salivary cortisol showed a power of 0.70, while the one of salivary testosterone was 0.87, and the one of perceived stress 0.24.

**Table 3:** AUC_G_ means and standard deviations

Outcome	TSST		GS-TSST	
	M (SD)	95% CI	M (SD)	95% CI
C	620.12 (70.19)	475.57/764.68	697.19 (76.52)	539.60/854.78
T	9935.58 (3289.98)	3159.75/16711.41	9596.66 (2724.32)	3985.81/15207.50
VAS	98.59 (11.18)	75.57/121.61	92.31 (12.37)	66.84/117.78

*M* mean, *SD* standard deviation, *95% CI* 95% confidence interval with lower and upper bound, *C* salivary cortisol, *T* salivary testosterone, *VAS* visual analogue scale assessing perceived stress

These findings contradict hypothesis 2, but indicate that the overall levels of salivary cortisol, testosterone, and perceived stress were similar among participants exposed to gay-related or general social stressors.

## Discussion

The study’s objective was to compare the stress response of gay men to general and gay-specific stressors. Our findings indicate that gay men react similarly to gay-related and general acute stressors induced by social stress paradigms. We did not find differences between the two conditions in stress reactivity—measured as the difference between the baseline stress level and the post-induction stress level—or in the overall level in stress outcomes throughout the whole session.

When descriptively comparing the salivary cortisol values with those reported for gay men in a previous study, we find that the gay men in our study showed slightly lower cortisol levels on average. Hatzenbuehler and McLaughlin (2014) reported in a table that gay men with a background of high structural stigma had roughly 9.4 nmol/L directly before the GS-TSST, around 10.4 nmol/L 5 min after the GS-TSST, and about 10.1 nmol/L 20 min after the GS-TSST. Gay men with low structural stigma had values of around 10.1, 13.4, and 12.2 nmol/L at these three time points, respectively (Hatzenbuehler and McLaughlin 2014). Note that the numbers are estimates based on a figure. Compared to these findings, gay men’s salivary cortisol in the GS-TSST condition of our study was lower at all three time points (compare Table 2). The fact that our participants had lower levels of salivary cortisol than both groups of American gay men may be indicative of a stronger hypocortisolism in our sample. This may be due to the current study’s sampling of gay men with at least one experience of gay-related discrimination.

When descriptively comparing the testosterone levels of our sample with a sample of men of any sexual orientation who were exposed to the TSST, we find that they had a higher baseline testosterone level of 112.8 pg/mL (Knight et al. 2017) than our sample (TSST condition: 82.68 pg/mL; GS-TSST condition: 94.53 pg/mL). Furthermore, they had testosterone levels of roughly 170 pg/mL directly before the TSST, of 170 pg/mL directly afterwards, and of about 135 pg/mL 20 min afterwards (Knight et al. 2017). As above, these values were estimated from a figure. When we compare these values with those of our participants (Table 2), our sample of gay men showed lower levels at all these time points in both the TSST and GS-TSST conditions, with the only exception being higher values in the TSST condition 20 min after the test, which was due to one outlier. Since gay men have been reported to have similar testosterone levels to heterosexual men (Burke and Bribiescas 2018; Zitzmann and Nieschlag 2001), these descriptive findings may be a further indicator of allostatic load in our sample.

A possible reason for these descriptive indicators of allostatic load and our null findings between the two stress-inducing conditions is that chronic gay-related stressors may proliferate and beget other forms of stressors that otherwise would not be encountered (LeBlanc et al. 2015). As such, it has been found that gay men experience general stressors more often than heterosexual men, such as early-life adversity, including physical and sexual abuse, homelessness, intimate-partner sexual violence (McLaughlin et al. 2012), debt, terminations of personal relationships (Przedworski et al. 2015), as well as general, not necessarily gay-related discrimination in contexts such as housing, employment/income, social relationships, and health care (Hatzenbuehler et al. 2013).

It is likely that the combination and possible proliferation of both types of stressors contributes to the increased risk of physical stress-related illnesses (Branström et al. 2016), mental disorders (Plöderl and Tremblay 2015; Ross et al. 2018; Semlyen et al. 2016), and increased mortality found in gay men compared to heterosexual men (Cochran et al. 2016). Therefore, treatments that aim to alleviate mental-health problems in gay men should work on gay-related as well as general factors. They can include teaching strategies on how to cope with different forms of stressors, such as building social support and assertiveness, and decreasing emotional dysregulation and rumination (Pachankis et al. 2015). An example of an intervention with such a broader scope is the compassionate-focused approach by Pepping et al. (2017), which trains gay men with regard to emotion regulation, compassion, and non-judgmental attention, in situations both of gay-related and general stress. Unfortunately, no data on the effectiveness of this approach are currently available. Besides interventions with gay men who are already suffering from high stress levels and the adverse consequences thereof, preventive strategies can be used, such as seminars on how to cope with or avoid different forms of stressors.

The development of interventions as well as prevention strategies regarding gay men’s mental health are highly relevant, since studies found that even though the social situation for LGB individuals has improved substantially within the last decades, minority-stress exposure has not changed from older to younger sexual-minority cohorts (Meyer et al. 2021), nor have mental-health measures significantly improved after access to same-sex marriage (Carpenter et al. 2021).

In addition, we are aware of only one prior study which compared the psychobiological stress response of LGB individuals to LGB-related and non-LGB-related stressors. They found that they had a stronger vascular response when exposed to an LGB-related TSST-like test, compared to an LGB-unrelated one (Rosati et al. 2021). These findings are contrary to our null findings and show that gay-related stressors may affect certain stress markers differently than others.

Several limitations of the present study should be mentioned. These include the relatively low number of participants and low power in some of our tests, which decreased the likelihood of finding significant and generalizable results. Moreover, we did not include a gay control group without experiences of gay-related stress or a heterosexual control group. A major advantage of our study lies in the experimental design, which ensured internal validity. Due to this and since all participants passed through both experimental conditions, it is unlikely that confounding variables (such as personality factors or self-efficacy expectations) influenced our data significantly. Furthermore, the inclusion of psychobiological outcome variables as well as a subjective one (perceived stress) was advantageous, as it not only broadened the study’s scope but also enabled an internal replication of our findings by comparing the stress response in terms of different outcomes. In addition, using the TSST and an analog gay-related version ensured internal and external validity, since the TSST is among the most robust stress-inducing tests that exist (Dickerson and Kemeny 2004) and has been found to be valuable when used with groups experiencing discrimination (Keenan et al. 2021).

As our study is one of the first to look at possible differences between the stress response to gay-specific and general stress, it can be considered a valuable stepping stone for future research. Future studies could examine potential differential stress outcomes in other stigmatized groups with reported altered HPA axis activity and hypocortisolism, such as racial/ethnic (Adam et al. 2015) and gender minorities (DuBois et al. 2017), as well as those with intersecting underrepresented identities (Cook et al. 2017). In addition, to learn more about non-socially evaluative forms of gay-related and general stressors that occur in everyday life, field studies are needed.

## Conclusion

Since gay men were found to respond similarly to gay-related and general stressors, minority-stress research as well as interventions with gay men should take general stressors into account. Furthermore, future research should investigate whether a proliferation from gay-related to general stressors exist.

## Data Availability

Data, material, and code are available from the corresponding author upon reasonable request.
